# Cross Sectional Study of the Community Self-Reported Risk of Obstructive Sleep Apnoea (OSA) and Awareness in Thessaly, Greece

**DOI:** 10.3390/clockssleep4010004

**Published:** 2022-02-10

**Authors:** Petros Kassas, Georgios D. Vavougios, Chrissi Hatzoglou, Konstantinos I. Gourgoulianis, Sotirios G. Zarogiannis

**Affiliations:** 1 Department of Respiratory Medicine, Faculty of Medicine, School of Health Sciences, University of Thessaly, BIOPOLIS, 41500 Larissa, Greece; pkassas@uth.gr (P.K.); gvavou@uth.gr (G.D.V.); chatz@med.uth.gr (C.H.); kgourg@med.uth.gr (K.I.G.); 2 Department of Physiology, Faculty of Medicine, Scholl of Health Sciences, University of Thessaly, BIOPOLIS, 41500 Larissa, Greece

**Keywords:** obstructive sleep apnea syndrome, OSAS awareness, sleep disorders, prevalence, epidemiology, Greece, telephone survey, Berlin questionnaire, Epworth Sleepiness Scale

## Abstract

The purpose of this study was to investigate the self-reported risk of obstructive sleep apnea syndrome (OSAS) in the municipality of Thessaly, Greece, and the level of awareness of both the disease and its diagnosis. Inhabitants of Thessaly (254 total; 84 men and 170 women) were studied by means of questionnaires via a telephone-randomized survey. This comprised: (a) the Berlin questionnaire for evaluation of OSAS risk; (b) the evaluation of daytime sleepiness by the Epworth Sleepiness Scale; and (c) demographic and anthropometric data. The percentage of participants at high risk for OSA was 26.77%, and the percentage of people who were at high risk of excessive daytime sleepiness was 10.63%. High risk for OSAS was found to be 3.94%. No significant differences were found between high- and low-risk OSAS participants associated with age, smoking and severity of smoking. Regarding the knowledge of the community about OSAS, the majority of the sample was aware of the entity (64.17%), while fewer had knowledge about the diagnosis (18.50%) and polysomnography (24.80%). The high risk of OSA prevalence and the low awareness of the diagnosis of OSA highlights the need for the development of health promotion programs aiming at increasing the disease awareness in the general population in order to address OSA more effectively.

## 1. Introduction

Obstructive sleep apnoea syndrome (OSAS), according to the WHO, is a clinical disorder with frequent breathing pauses during sleep, usually accompanied by snoring [1]. These apnea and hypopnea’s recurrent episodes are due to complete or partial occlusion of the upper airway during sleep [2]. The standard diagnostic test through which obstructive sleep apnoea syndrome diagnosis is established is the Polysomnography (PSG) that involves a comprehensive sleep evaluation recording several aspects of body activity during sleep [3]. More specifically, a diagnosis of OSAS is established either when a patient has over five apneas–hypopneas per hour of sleep (Apnea–Hypopnea Index, AHI) and associated symptoms (e.g., excessive daytime sleepiness, fatigue or impaired cognition), or has OSA due to an AHI of 15 or more, regardless of the associated symptoms [3]. According to the latest clinical practice guidelines for diagnostic testing for adult OSAS, the use of questionnaires is strongly discouraged for diagnosis without a subsequent PSG [4]. However, the use of self-reported questionnaires such as the Berlin Questionnaire (BQ), indicating high risk for OSAS, is necessary for community screening and subsequent referral to sleep clinics [5].

OSAS is an underdiagnosed chronic disease. Untreated patients are at a significantly increased risk of developing cardiovascular, metabolic and neurocognitive diseases, as well as motor vehicle (MVAs) and/or work accidents [6]. Additionally, OSA is strongly associated with inflammation disorders and, interestingly, with allergic and vasomotor rhinitis [7]. Studies from the USA, Australia, India, China and Korea report that the prevalence in the general adult population spans from 3 to 7% in men, and 2 to 5% in women, to more than 49% depending on age and gender [8,9].

## 2. Results

### 2.1. Sample Characteristics and Responsiveness Rate

**Figure 1:**
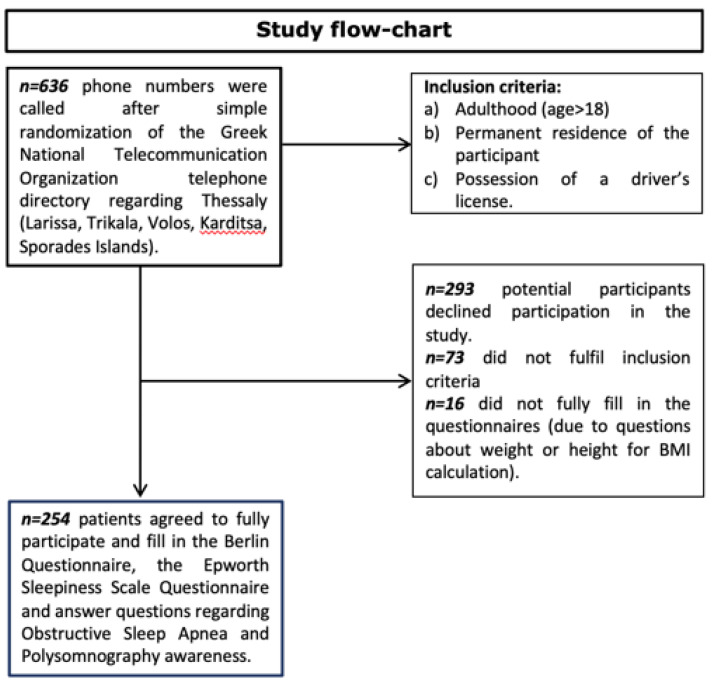
Flow-chart demonstrating study design.

In this study 254 people from the prefecture of Thessaly participated (84 males and 170 females, mean age 54.11 ± 16.13, mean BMI 26.72 ± 5.05) by means of questionnaire completion via a telephone-randomized survey. The geographical distribution of participants was as follows: 56 from Larissa, 50 from Volos, 48 from Karditsa, 50 from Trikala, and 50 from the Sporades insular complex. A study flow chart is presented in Figure 1. Detailed characteristics of the participants are shown in Table 1.

### 2.2. OSAS Risk According to BQ and ESS

Out of all the participants, 68/254 (26.77%) were found to be high risk in the BQ. Gender wise, 25/84 (29.76%) males and 43/170 (25.29%) females were found to be high risk. Regarding ESS, 27/254 (10.63%) participants scored above 10 in the ESS, 10/84 (11.90%) males and 17/170 (10%) females. The participants that were found to be high risk, both in BQ and ESS, and were thus considered at high risk for OSAS, were 10/254 (3.94%) overall; 5/84 (5.95%) males and 5/170 (2.94%) females. Detailed descriptions of the self-reported community prevalence of OSA, EDS and OSAS in our sample are shown in Table 2. There were no significant differences regarding the frequencies of the participants at high risk among the five different geographical areas (*p* = 0.79 regarding BQ, *p* = 0.72 regarding ESS, *p* = 0.87 regarding BQ and ESS). In all cases, male participants were slightly, but not significantly, at higher risk in BQ (*p* = 0.46), in ESS (*p* = 0.67) and in their combination (*p* = 0.31).

### 2.3. Participants’ Awareness Regarding OSAS and Its Clinical Diagnosis

Participants’ awareness was assessed by a set of three questions. The first question referred to their knowledge about the existence of OSAS; the second was about the way the syndrome is diagnosed; the third regarded whether the participants knew what a PSG Sleep Study is. Out of 254 participants, 163 (163/254; 64.17%) answered that they knew what OSAS is, 47/254 (18.50%) were aware of how the diagnosis of OSAS is established, and 63/254 (24.80%) answered that they knew what a PSG Sleep Study is. In Table 3, the frequency of answers to these three questions are presented per city. There were no significant differences regarding the frequencies of answers among the five different geographical areas (*p* = 0.08 regarding OSAS awareness, *p* = 0.51 regarding the OSAS diagnosis, *p* = 0.48 regarding PSG awareness). In all cases, male participants were slightly, but not significantly, more aware than female participants regarding OSAS (*p* = 0.27), its diagnosis (*p* = 0.61), and PSG (*p* = 0.76).

## 3. Discussion

In this study, we report novel findings on community self-reported OSAS prevalence in the mainland of Greece. Based on the latest census of 2011, the population of Thessaly is 732.762 people. A sample of 254 people from the general population from four cities (Larissa, Volos, Trikala, Karditsa) and an insular complex (Sporades) of the Thessaly prefecture in Greece showed that 26.77% of the population are at high risk of OSA. Our results are close to the prevalence reported in the USA (26%) and Norway (24.3) [10,11]. There is a scarcity of data regarding the community self-reported OSA prevalence in the countries of the Mediterranean. In the only study that could serve as a comparison in this context, because it was conducted in Cyprus with population of Greek origin, the results showed a percentage of 34% being at high risk of OSA. Although the sample was far greater than ours (4118 participants), the assessment tool was a different questionnaire, STOP-Bang, which is far more sensitive than BQ. This could account for the nearly 10% difference between the two studies [12,13].

Other studies that involve community OSA prevalence in Mediterranean countries come from Lebanon [14], and Morocco [15]. In the first one, involving 501 participants with comparable age (45.2 ± 15.2 years) and male percentage (36%) with our study, 31.3% were found to be at high-risk of OSA, based on BQ results [14]. In the second study, involving 503 Moroccan participants with similar age (42.7 ± 14.1 years) to our study, the OSAS prevalence was far lower, specifically around 9.5% [15]. Although this is a speculation, differences in smoking, obesity, and genetic profiles between the three populations could account for the differences in percentages found to be at high risk of OSA.

As far as daytime sleepiness is concerned, the ESS results of our study showed a self-reported prevalence of 10.63%. This result is higher than the study from Norway (8.9%) [11], lower than Sweden and Iceland (13.1%) [16], and much lower than Canada (33%) [17].

Regarding the prevalence of OSAS studies, the USA report percentages from 6% to 17% [18,19], and in Brazil 32.8% [20]. The gender-specific prevalence for males ranged from 3.1% to 49.7% [9,20,21,22,23,24,25,26], and for females from 1.2% to 30.5% [9,20,21,22,24,25,26]. In the majority of the published studies about OSAS, PSG was the method for establishing diagnosis. In our study, the self-reported OSAS prevalence (participants being at high risk in both BQ and ESS) was found to be 3.94% (men: 5.95%; women: 2.94%). Thus, our results could be deemed comparable to the reported literature, bearing in mind that PSG is a more sensitive method for OSAS diagnosis. These results provide important information given that self-reported snoring in patients assessed with BQ, and found to have OSA with established cardiovascular disease, are associated with higher risks of stroke, irrespective of receiving treatment [27]. This is even more important should one consider the fact that therapeutic choice made in treating OSA could vary, depending on economic resources [28].

Finally, although the study population were aware of the OSAS entity to a relatively high percentage (64.17%), a far smaller percent were aware of the method of diagnosis (19.12%) and PSG more specifically (25.98%). This is an alerting finding that, on the other hand, shows that a lot can be improved with focused health promotion interventions aiming more informing at the community about OSAS and its diagnosis.

A limitation of our study was the use of questionnaire tools instead of PSG, which is the gold standard for OSAS diagnosis [4]. Another limitation was the use of ESS and BQ instead of other questionnaires with higher sensitivity to detect OSAS, such as the STOP-Bang questionnaire [29]. However, since we performed a telephone survey, we would have had to rely on non-standardized self-reported neck circumference measurements, that would potentially compromise the results of the questionnaire. Indeed, STOP-Bang is the preferred tool for OSAS detection in sleep clinics of low-resource countries where PSG is not available [30]. In a setting such as ours, however, the use of BQ as an epidemiological tool in the community is justified, especially for ruling out severe OSAS, despite the inherent limitations of sleep-related questionnaires [29,30,31,32].

## 4. Materials and Methods

### 4.1. Study Protocol

The selection of the subjects was performed in a randomized way via the internet telephone directory of the National Telecommunication Organization (OΤΕ—https://www.11888.gr/white-pages/; last assessed on 6 October 2017) following a standardized pattern by choosing one telephone number for every fifty telephone numbers. The simple randomization technique of flipping a coin was followed (heads we called the number; tails we did not call the number) in every case [33]. Cell phones enlisted were excluded in order to assure that the participants were permanent residents. Initially, the subjects were introduced to the scope and methodology of the study, and if they wished to participate, they provided their informed consent prior to the commencement of the questionnaire completion. Research conductors had to ensure every participant was over 18 years of age and had a driver’s license. Every questionnaire had to be fully answered to be qualified for further analysis. Thus, the inclusion criteria for the participants to be eligible to enroll in the study were: (a) adulthood (age > 18); (b) permanent residence of the participant; and (c) possession of a driver’s license. Out of the 636 phone calls that were carried out, 254 responders volunteered to participate in our study providing informed consent (responsiveness percentage: 39.9%). A flow chart of the study design is presented in Figure 1. The study sample comprised 84 men and 170 women. Of the sample, 48 participants were from Karditsa, 50 from Trikala, 50 from Volos, and 56 from Larissa. The research protocol was approved by the Research Committee of the University of Thessaly (protocol number 2800/2017).

### 4.2. Assessment Tools

The assessment tools which were used for the purposes of the research were: (a) the BQ for evaluation of OSAS risk; (b) the Epworth Sleepiness Scale (ESS) for the evaluation of daytime sleepiness; and (c) an awareness regarding the disease and its diagnosis and demographic with the anthropometric data questionnaire. Both versions of the BQ and ESS used were translated and adjusted to Greek [34,35].

### 4.3. Statistical Analysis

Data were tabulated in an Excel spreadsheet and statistical analysis was performed with Graphpad Prism v.9.0 using the chi-square (when frequencies were compared) and the Student’s unpaired *t*-test (when demographic variables were compared) where appropriate. A *p* < 0.05 was considered statistically significant.

## 5. Conclusions

The high prevalence of OSA and the low awareness of the way it is diagnosed highlights the need for development of Health Promotion programs aiming at increasing the disease awareness in the general population. Moreover, there is a need for primary care healthcare professionals to be continuously educated towards the early diagnosis and therapy of OSA in order to reduce the burden of the disease in Greece.

## Figures and Tables

**Table 1:** Characteristics of the participants of the study.

	Males (84/254; 33.07%)	Females (170/254; 66.93%)	*p* Value
Age (years)	54.12 ± 17.00	54.11 ± 15.74	>0.05
BMI (kg/m²)	27.66 ± 4.79	26.25 ± 5.13	0.036
Smokers (%)	53.73	32.12	<0.05

**Table 2:** High-Risk Prevalence of participants for OSA, EDS and OSAS.

	BQ High Risk		ESS High Risk		BQ and ESS High Risk	
Gender	M (#)	F (#)	M (#)	F (#)	M (#)	F (#)
Larissa	7	5	1	3	0	1
(*n* = 56)						
Volos	4	9	1	5	0	2
(*n* = 50)						
Trikala (*n* = 50)	5	8	3	3	2	0
Karditsa (*n* = 48)	5	9	4	3	2	0
Sporades (*n* = 50)	4	12	1	3	1	2
Total (*n* = 254)	25	43	10	17	5	5
% over total per gender	29.76%	25.29%	11.90%	10.00%	5.95%	2.94%
% over total	26.77%		10.63%		3.94%	

**Table 3:** Participants’ Awareness regarding OSAS, its diagnosis’ and PSG per insular complex.

	Awareness Regarding OSAS		Awareness Regarding OSAS Diagnosis		Awareness Regarding PSG	
Gender	M (#)	F (#)	M (#)	F (#)	M (#)	F (#)
Larissa (*n* = 56)	7	20	2	5	5	9
Volos (*n* = 50)	17	16	3	6	4	6
Trikala (*n* = 50)	11	24	5	6	5	12
Karditsa (*n* = 48)	13	20	4	8	6	6
Sporades (*n* = 50)	10	25	3	5	2	8
Total (*n* = 254)	58	105	17	30	22	41
% over total per gender	69.05%	61.76%	20.24%	17.65%	26.19%	24.12%
% over total	64.17%		18.50%		24.80%	

## Data Availability

Data are available on reasonable request from the corresponding author.
